# Suicidal behavior among inpatients with severe mental conditions in a public mental health hospital in Uganda

**DOI:** 10.1186/s12888-023-04858-x

**Published:** 2023-05-19

**Authors:** Joan Abaatyo, Alain Favina, Steven Elijah Bulega, Mark Mohan Kaggwa

**Affiliations:** 1 Department of Psychiatry, Faculty of Medicine, Mbarara University of Science and Technology, Mbarara, Uganda; 2 Faculty of Medicine, Mbarara University of Science and Technology, Mbarara, Uganda; 3 Department of Psychiatry and Behavioural Neurosciences, McMaster University, McMaster, Canada

**Keywords:** Suicidal behaviors, Suicidal attempt, Suicide, Financial constraints, Depression, Substance use, Severe mental conditions, Uganda

## Abstract

**Background:**

Suicidal behaviors are prevalent among inpatients with severe mental conditions and may result in many dying by suicide. Few studies have focused on the burden of suicidal behaviors among these inpatients in low-income settings, despite suicide being consistently higher in lower-income countries such as Uganda. This study, therefore, provides the prevalence and associated factors of suicidal behaviors and suicide attempts among inpatients with severe mental conditions in Uganda.

**Method:**

This was a retrospective chart review of all individuals admitted with severe mental conditions to a large psychiatry inpatient unit in Uganda for four years (2018–2021). Two separate logistic regressions were conducted to determine the factors associated with suicidal behaviors or suicidal attempts among the admitted individuals.

**Results:**

The prevalence of suicidal behavior and suicidal attempts among 3104 (mean age = 33, Standard deviation [SD] = 14.0; 56% were males) were 6.12% and 3.45%, respectively. Having a diagnosis of depression increased the likelihood of both suicidal behaviors (adjusted odds ratio [aOR]: 5.36; 95% confidence interval [CI]: 2.14–13.37; p =0.001) and attempts (aOR: 10.73; 95% CI: 3.44–33.50; *p* < 0.001). However, a diagnosis of substance-related disorder increased the likelihood of having attempted suicide (aOR: 4.14; 95% CI: 1.21–14.15; p = 0.023). The likelihood of having suicidal behavior decreased as one increased in age (aOR: 0.97; 95% CI: 0.94–0.99; p = 0.006) and increased among individuals reporting stress from financial constraints (aOR: 2.26; 95% CI: 1.05–4.86; p = 0.036).

**Conclusion:**

Suicidal behaviors are common among inpatients managed for severe mental health conditions in Uganda, especially those with substance use and depressive disorders. In addition, financial stressors are a main predictor in this low-income country. Therefore, regular screening for suicide behaviors is warranted, especially among individuals with depression, and substance use, among those who are young, and among those reporting financial constraints/stress.

## Introduction

Suicide accounts for approximately 700,000 deaths annually [1, 2], with the majority of suicide being among individuals with mental health conditions, compared to the general population [3]. Some of the countries with the highest suicide rates are low and middle-income countries [4], yet, in these countries like Uganda, where the suicide rate was estimated to be 10.3 per 100,000 population in 2016 [5], few patients disclose suicidal behaviors (a major risk factor for suicide) to their clinicians due to stigma, overcorrection, criminalization, and loss of autonomy associated with the behaviors [6–10].

Suicidal behaviors manifest in various ways, including verbalizing suicidal thoughts, engaging in self-harm, writing about suicide or death, or making specific plans for suicide [11, 12]. Individuals who engage in suicidal behaviors are at an increased risk of suicide [10, 13, 14]. For this fact, various scholars have recommended significant attention to the problem, especially among individuals with severe mental illness – who have a proportionally higher prevalence of and stronger associations with suicide and suicidal behaviors [15–17]. Approximately 10% of individuals with mental health problems die by suicide, and almost 90% of those who die by suicide (in general) have a mental disorder diagnosis [18, 19]. For example, more than 50% of individuals who die by suicide experience symptoms of depression [20], and in Uganda, five (three with depression) out of 23 university students who died by suicide had a mental illness [6]. Various other risk factors for suicidal behavior have been documented among individuals with mental health conditions, including; a history of emotional and physical trauma or abuse, access to lethal means, male gender, young age, unemployment, family history of mental disorder, family history of suicide, and a lack of regular contact with a significant other [21–25]. In addition, individuals having a suicidal attempt are at higher risk of dying by suicide than those with other forms of suicidal behaviors [16]. Thus, properly managing and supporting individuals with suicidal attempts is crucial in suicide prevention [26].

Suicide is a preventable cause of death, and its prevention requires early identification and intervention of people at most risk, like those diagnosed with severe mental conditions [27]. Individuals with mental illness conditions often have complex needs that require specialized interventions. A better understanding of the specific factors contributing to suicidal behavior in this population can inform the development of targeted and effective interventions. By understanding the factors that contribute to suicidal behaviors, researchers can develop targeted and evidence-based interventions that can save lives. This study, therefore, assesses the factors associated with suicidal behaviors and suicide attempts among inpatients with severe mental conditions in Uganda.

## Methods

This was a retrospective chart review of 3104 (mean age = 33, SD = 14.0; 56% males) individuals with severe mental conditions admitted to a large psychiatry inpatient unit in Uganda between 2018 and 2021. At this unit, patient information is captured by clinicians (i.e., psychiatric clinical officers, psychiatry residents, and psychiatrists), who make the diagnosis based on the Diagnostic and Statistical Manual of Mental Disorders, Fifth Edition (DSM V). However, no standardized tool is used in capturing clinical data.

The study was approved by the institution’s ethics committee (MUST-2021-229). The following information was extracted from the individuals’ admission charts: (i) age, (iv) gender, (ii) marital status, (iii) level of education, (iv) employment status, (v) history of the different stressors present – a predesigned list was used to cross-check the stressors in the patient’s chat/file, (vi) current suicidal behavior(s), (vii) current suicide attempts, (viii) final diagnosis on discharge, and (ix) year of admission (pre-COVID pandemic vs. during the COVID-19 pandemic). By specifying the year as pre vs. during COVID, we aimed to capture any potential differences in the prevalence or patterns of suicidal behavior among individuals with severe mental conditions before and after the onset of the pandemic. This was because the pandemic limited access to healthcare and support services and caused significant stress and anxiety for many individuals, particularly those with pre-existing mental health conditions [28].

Data were captured by five pairs of trained research assistants with medical training and worked under the authors’ supervision. All research assistants had finished medical school training and were certified in responsible conduct of research. The authors with experience in hospital-based records review provided training and supervision. Each inpatient file was reviewed by two individuals independently. Any potential inconsistencies were rectified following a discussion with the two conflicting research assistants and the supervisor responsible for that specific period. The final edits were made to the first entry, and the corresponding version was deleted. For duplicates, they were automatically removed using Microsoft Excel.

### Outcome variables

Suicidal behaviors included individuals with current suicidal ideations, thoughts, plans, attempts, and unspecified suicidal behaviors. This was captured as a “*Yes”* or “*No”* response for their presence.

Suicide attempt – Due to the clinical importance of suicide attempts and its strong link to dying by suicide, this was considered a secondary outcome variable. Individuals with the presence of a recent suicide attempt(s) prior to admission were recorded with a “*Yes*” and “*No*”.

### Data analysis

Data were cleaned in Microsoft Excel, and analysis was done in STATA version 15.0. This study presented categorical variables using frequencies and percentages, while continuous variables were presented using means and standard deviations or medians and interquartile ranges. Two separate logistic regressions were conducted to determine the factors associated with suicidal behaviors or suicidal attempts among the admitted individuals. This study’s significance level was set at less than 5% for a 95% confidence interval.

## Results

### Suicidal behaviors

The prevalence of suicidal behavior was 6.12% (n = 190/3104). The mean age of those with suicidal behavior was 31.2, *SD* = 12.5. The likelihood of having suicidal behavior decreased with increasing age (aOR: 0.97; 95%CI: 0,94-0.99; *p* = 0.006). However, stress from financial constraints increased the likelihood of having suicidal behavior (aOR: 2.26; 95%CI: 1.05–4.86; *p* = 0.036). Compared to Bipolar Affective Disorder (BAD), having a depressive disorder increased the likelihood of having suicidal behavior (aOR: 5.36; 95% CI: 2.14–13.37; *p* = < 0.001) (Table 1).

**Table 1:** Demographic and clinical characteristics and logistic regression showing factors associated with having suicidal behaviors among individuals admitted to the psychiatry unit

Variable	Suicidal Behavior n (%) 190 (6.12)	Bi variable analysis		Multivariable analysis	
		Crude Odds ratio (95% confidence interval)	*p*-value	Adjusted odds ratio (95% confidence interval)	*p*-value
**Age** (mean, *SD)*	31.2 (12.5)	0.98 (0.97–0.99)	**0.010**	0.97 (0.94–0.99)	**0.006**
**Number of days with symptoms before admission** (Median, *IQR)*	7 (3–21)	1.00 (0.85-1.00)	0.190		
**Number of days on the ward** (median, *IQR)*	5 (3–10)	1.00 (0.99–1.01)	0.764		
**Gender**					
Female	79 (41.6)	1 (reference)	0.482		
Male	111 (58.4)	1.11 (0.82–1.50)			
**Positive family history**	39 (20.5)	1.13 (0.79–1.63)	0.505		
**Marital status**					
Married /cohabiting	75 (46.0)	1 (reference)			
Divorced	1 (0.6)	0.39 (0.05–2.88)	0.356		
Separated	7 (4.3)	0.51 (0.23–1.13)	0.099		
Single	76 (46.6)	1.45 (0.83–1.60)	0.409		
Widowed	4 (2.5)	0.67 (0.24–1.88)	0.446		
**Level of education**					
None	31 (23.5)	1 (reference)		1 (reference))	
Primary	35 (26.5)	1.15 (0.70–1.89)	0.577	1.26 (0.52–3.05)	0.616
Secondary	40 (30.3)	1.72 (1.06–2.79)	**0.028**	1.76 (0.72–4.31)	0.217
Tertiary	26 (19.7)	1.72 (1.00-2.94)	0.050	1.85 (0.74–4.63)	0.187
**Employment status**					
Unemployed	97 (63.4)	1 (reference)			
Employed	56 (36.6)	0.82 (0.50–1.34)	0.438		
**Year**					
Pre-covid	106 (56.1)	1 (reference)			
During covid	83 (43.9)	0.78 (0.58–1.05)	0.102		
**Type of stressor present**					
Family and/or marital conflicts (yes/no)	32 (16.8)	2.17 (0.45–3.24)	**< 0.001**	1.37 (0.74–2.51)	0.305
Financial constraint (yes/no)	17 (18.3)	2.60 (1.46–4.63)	**0.001**	2.26 (1.05–4.86)	**0.036**
Other stressors (yes/no)	19 (10.0)	2.38 (1.44–3.94)	**0.001**	1.37 (0.70–2.67)	0.361
**Diagnosis**					
Bipolar Affective Disorder	29 (15.9)	1 (reference)		1 (reference)	
Depressive disorders	55 (30.2)	12.18 (6.27–23.64)	**< 0.001**	5.36 (2.14–13.37)	**< 0.001**
Schizophrenia spectrum and other psychotic disorders	52 (28.6)	1.49 (0.77–2.88)	0.237	1.04 (0.44–2.43)	0.937
Substance-related disorders	18 (9.9)	2.98 (1.34–6.62)	**0.007**	1.89 (0.66–5.42)	0.232
Other disorders**	28 (15.4)	2.51 (1.21–5.21)	**0.014**	1.64 (0.64–4.34)	0.304

*Other stressors = death of a loved one, sexual abuse, loss of a job, having been robbed

**Other disorders = Neurocognitive disorders, Somatic symptom disorders, Anxiety disorders, Acute stress disorder, conduct disorder, Medication induced movement disorders

### Suicidal attempt

The prevalence of suicidal attempts was 3.45% (n = 107/3104). The mean age of those with suicidal behavior was 32.5, *SD* = 13.1. Compared to BAD, having a depressive disorder (aOR: 10.73; 95% CI: 3.44–33.50; *p* < 0.001) and a substance-related disorder increased the likelihood of having attempted suicide (aOR: 4.14; 95% CI: 1.21–14.15; *p* = 0.023) (Table 2).

**Table 2:** Demographic and clinical characteristics and logistic regression showing factors associated with suicide attempts among individuals admitted to the psychiatry unit

Variable	Suicidal attempt n (%) 107 (3.45)	Bivariable analysis		Multivariable analysis	
		Crude Odds ratio (95% confidence interval)	p-value	Adjusted odds ratio (95% confidence interval)	p-value
**Age (**mean, *SD)*	32.5 (13.1)	0.99 (0.98-1.00)	0.335		
**Number of days with symptoms before admission (**Median, *IQR)*	7 (2.5–21)	0.99 (0.99-1.00)	0.966		
**Number of days on ward** (median, *IQR)*	5.5 (3–11)	1.00 (0.99–1.02)	0.892		
**Gender**					
Female	43 (40.2)	1 (reference)	0.417		
Male	64 (59.8)	1.18 (0.79–1.74)			
**Positive family history**	22 (20.6)	1.13 (0.70–1.82)	0.619		
**Marital status**					
Married /cohabiting	48 (50.5)	1 (reference)			
Divorced	1 (1.1)	0.62 (0.08–4.64)	0.644		
Separated	4 (4.2)	0.47 (0.17–1.31)	0.147		
Single	40 (42.1)	0.93 (0.61–1.43)	0.754		
Widowed	2 (2.1)	0.52 (0.13–2.19)	0.376		
**Level of education**					
None	19 (26.0)	1 (reference)			
Primary	19 (26.0)	1.01 (0.53–1.93)	0.969		
Secondary	23 (31.5)	1.58 (0.85–2.94)	0.144		
Tertiary	12 (16.4)	1.26 (0.60–2.62)	0.542		
**Employment status**					
Unemployed	76 (86.4)	1 (reference)	0.664		
Employed	12 (13.6)	0.87 (0.47–1.62)			
**Year**					
Pre-covid	54 (50.5)	1 (reference)			
During covid	53 (49.5)	0.99 (0.68–1.46)	0.979		
**Type of stressor present**					
Family and/Marital conflicts (yes/no)	18 (16.8)	2.10 (1.24–3.52)	**0.005**	1.10 (0.57–2.13)	0.771
Financial constraint (yes/no)	11 (20.0)	2.79 (1.38–5.61)	**0.004**	2.13 (0.98–4.62)	0.056
Other stressors (yes/no)	11 (10.3)	2.37 (1.24–4.52)	**0.009**	1.28 (0.62–2.64)	0.503
**Diagnosis**					
Bipolar Affective Disorder	13 (12.5)	1 (reference)		1 (reference)	
Depressive disorders	33 (31.7)	12.18 (6.27–23.64)	**< 0.001**	10.73 (3.44–33.50)	**< 0.001**
Schizophrenia Spectrum and Other Psychotic Disorders	29 (27.9)	1.49 (0.77–0.88)	0.237	1.82 (0.59–5.63)	0.299
Substance-related disorders	12 (11.5)	2.89 (1.34–6.62)	**0.007**	4.14 (1.21–14.15)	**0.023**
Other disorders**	17 (16.4)	2.51 (1.21–5.21)	**0.014**	2.85 (0.87–9.36)	0.083

*Other stressors = death of a loved one, sexual abuse, loss of a job, having been robbed

**Other disorders = Neurocognitive disorders, Somatic symptom disorders, Anxiety disorders, Acute stress disorder, conduct disorder, Medication induced movement disorders

## Discussion

The present study investigated the prevalence and associated factors for suicidal behavior and suicidal attempts among individuals with severe mental conditions admitted to a psychiatric unit in Uganda between 2018 and 2021. The prevalence of suicidal behavior was approximately twice that of suicidal attempt (i.e., 6.12% vs. 3.45%). Having a depressive disorder was associated with an increased likelihood of having both suicidal behavior and suicidal attempt. An increase in age reduced the likelihood of having suicidal behavior while having financial constraints increased the likelihood of suicidal behavior. Having substance-related disorders was associated with increasing the likelihood of suicidal attempt.

The prevalence of suicidal behavior in this study was lower than 28.6% which was reported by a 2014 Ethiopian cross-sectional study among 385 outpatients living with mental illness based on the Suicide Behavioral Questionnaire-Revised [29]. The difference may be due to the Ethiopian study using a psychometric tool that provides standardized measures, which can help reduce subjectivity and improve the accuracy and reliability of the assessment. However, the present study had a large sample size than the Ethiopian study and was powered enough to accurately approximate the prevalence. Additionally, the duration, period, and location in which the two studies were conducted would also have contributed to the difference in the prevalence since sociopolitical factors vary across study periods. Thus, the present study controlled for such events due to a long-time span than the study in Ethiopia. In addition, the difference may be due to the fact that few patients in Uganda disclose their suicidal behaviors to clinicians due to stigma, overcorrection, criminalization, and loss of autonomy associated with the behaviors [6–8, 30]. Furthermore, in Uganda, it is not standard practice for healthcare professionals in healthcare facilities, including mental health facilities, to conduct screenings and evaluations for suicidal behavior, leading to lower rates [8, 31].

The prevalence of suicidal attempts in the present study was lower than 19% which was reported by a study among 18,018 individuals with mental health conditions attending 141 private hospitals in the USA [32]. The difference in the study results may be due to the larger sample size and the USA study reporting suicide attempts six months prior to admission [32]. The present study only reported current suicide attempts (prior to admission), which could have underreported the prevalence of suicide attempts among the admitted patients. Also, the difference may be due to significant differences in various cultural and socio-economic factors in the study settings, including stronger cultural and family support systems and less widespread access to lethal means in Uganda compared to the United States. Additionally, limited access to mental health services in Uganda [33, 34] compared to the USA could result in inadequate surveillance of patients and missed warning signs, hence the lower rates found.

A high level of family support complements care at the psychiatry facility in Uganda to the extent that patients stay in the hospital with their family members during admission [35]. This shows how supportive the family structure is in the patients’ lives and may be the reason for the low prevalence of suicidal behaviors. However, a prevalence of 6.12% for suicidal behavior and 3.45% for suicidal attempt among psychiatric in-patients is still considered high. A possible explanation for this high prevalence is that psychiatric patients experience many complex issues caused by mental illness and other factors. These range from the variability in symptoms of the psychiatric conditions like feelings of hopelessness in depression [36] and command hallucinations in schizophrenia [37], to social and economic factors, such as poverty, unemployment, isolation, limited access to resources and support, and stigma [38, 39].

Similar to other studies among patients with mental health conditions [21, 25, 29], the present study found that an increase in age was associated with a decreased likelihood of having suicidal behaviors. The protective effect of age may be due to several factors, including greater life experience and coping skills, stronger social support networks, and a greater sense of purpose and meaning in life [40]. Additionally, as one gets older, they may be less likely to engage in impulsive or reckless behavior, which can reduce their risk of suicide [41]. Additionally, the onset of many major mental health conditions illness starts at younger age, and the stress of dealing with a new life-changing diagnosis is thus higher at this age, contributing to feelings of hopelessness and suicidality [42]. However, despite our findings, suicide and suicidal behaviors have been noticed to be prevalent among the elderly population and are a global concern. Therefore, the protectiveness of increasing age may not be throughout the whole life span since the current study population had fewer older individuals records reviewed.

This study found that stress from financial constraints was associated with an increased likelihood of having suicidal behaviors, a finding consistent with various reports like that among Korean adults by Park et al. [43] and a study among Ugandan university students which found that financial stress, in terms of failure to pay tuition fees increased the odds of suicidal behaviors [9]. The possible explanations for this finding include financial stress can arise from a variety of factors, including unemployment, underemployment, and poverty, which can lead to feelings of hopelessness, helplessness, and despair that can contribute to suicidal behaviors [4, 44]. Individuals with financial stress may also have limited access to resources such as mental health services, which can exacerbate their mental health problems and increase their risk of suicidal behaviors [45]. Financial stress can also cause or exacerbate mental health conditions, such as depression and anxiety, known risk factors for suicide [46]. Financial stress is just one of many factors that can contribute to suicidal behavior, and it is a complex issue that various factors can influence. Therefore, it is important to consider a holistic approach when addressing suicidal behavior among psychiatric in-patients experiencing financial stress.

This study found that having depressive disorders, when compared to bipolar affective disorders, was associated with an increased likelihood of having suicidal behaviors and suicidal attempts. This finding was congruent with that done by Radomski et al. (1999) at a university-based psychiatric treatment facility, which found that depression increases likelihood of all suicidal behavior compared to other psychiatric diagnoses [21]. In a study among Ugandan school going children and adolescents, the severity of depression increased the odds of having suicidal ideations [47]. A similar relationship was observed among university students in Uganda during the COVID-19 pandemic [48]. Depression is characterized by symptoms such as sadness, loss of interest or pleasure, feelings of worthlessness, and difficulty concentrating [49, 50]. Despite the strong relationship between suicidal behaviors and depression, suicidal behaviors are common among individuals living with all types of mental health conditions. Thus, individuals with mental health conditions in Uganda are believed to die more from suicide [51].

This study found that having substance-related disorders, when compared to BAD, was associated with an increased likelihood of attempting suicide. This finding was similar to a prospective Danish-nationwide study among individuals with mental health conditions, that found substance use disorders were strongly associated with risk of suicide attempts in people with severe mental health conditions [52]. In Uganda, several studies have reported a relationship between suicidal behaviors and use of substances [6, 53–55]. Many substances of abuse cause disinhibition and a tendency to act quickly on urges or in response to stimuli resulting in poor judgement, impulsivity and/or aggression [56]. The anger, poor judgement, and impulsivity synergistically contribute to an increase in suicide attempt due to the quick urge to act on triggers [57]. Substance-related disorders, such as alcohol and drug dependence, are often associated with a variety of negative consequences, including physical and mental health problems, social and economic difficulties, and legal problems [58, 59], which can lead to feelings of hopelessness and despair and resultant suicidal attempts. Substance use can also worsen symptoms of mental health conditions, such as depressive disorders, which are known risk factors for suicide.

### Limitations

The results of the present study should be interpreted with caution in view of the following limitations. First, this study was limited in scope by the relatively narrow range of the social, demographic, and clinical correlates, or risk factor variables that could be obtained from review of health management information system records of patients and missing data are not uncommon in such study designs. Additionally, chart reviews are likely to have missing diagnoses, differences in diagnostic patterns by providers, and the likelihood that sociodemographic/family history information might be missing for variety of reasons (e.g., not discussed with patient). For these reasons, we were unable to properly assess variables that were more intricate like; age at illness onset, family history of suicide, adherence to medication, adverse childhood events, family environment and extent of substance abuse/dependence. Second, in this study, we based our diagnosis on the medical evaluation that was done when patients were admitted, thus not capturing history of suicidal behaviors. It is necessary to repeat our findings using a more standardized and structured method of diagnostic interviews and capture history of these behaviors to clearly show a pattern of suicidal behaviors. Third, our analysis included data from only one location, which makes it difficult to apply the results to other places across the country. While the group of patients with suicidal behavior and attempted suicide in our study may not be representative of the entire population of Uganda, it is likely that the number of cases is actually higher, as it is unknown how many cases go unreported or receive no formal treatment. However, it should be noted that even among those who receive formal medical treatment, only a small percentage are treated at the type of specialized medical centers where this study was conducted. Some patients may receive treatment at primary care clinics, secondary care hospitals or private medical facilities. Finally, the study identified that those having depression, substance use, financial difficulties are at increased odds of having suicide risk. This is expected as the sample was selected from a group of individuals with potentially low-income earnings who tend to attend government facilities more. Yet, one of the indications for hospitalization suicide risk associated with diagnosis of depression, and substance use might be associated with low-income, thus the findings in this study. A large multicenter study with various study populations and having various subgroup analysis may be helpful in understanding suicidal behavioural phenomena.

## Conclusions

Suicidal behaviors are common among inpatients managed for mental health conditions in Uganda, especially those with substance use and depressive disorders. Half of those with suicidal behaviors have suicidal attempts. In this low-income country, financial stressors are a main predictor but as people age, the risk of suicidal behaviors reduces. Constant screening for suicide behaviors is warranted especially among individuals with depression, substance use, and reporting financial constrains/stress. However, multisector approaches are needed to tackle the problems since many facets are involved.

## Data Availability

The datasets will be made available to appropriate academic parties on request from the AJ.

## Abbreviations

HMIS: Health Management Information Systems
MRRH: Mbarara Regional Referral Hospital
